# Morphological and Molecular Characterization of *Hoplolaimus tuberosus* n. sp. (Nematoda: Hoplolaimidae) Associated with Potato in Uganda

**DOI:** 10.2478/jofnem-2025-0025

**Published:** 2025-07-19

**Authors:** Rose Mwesige, Joseph Maosa, Marjolein Couvreur, Wim Bert

**Affiliations:** Nematology Research Unit, Department of Biology, Ghent University, K.L. Ledeganckstraat 35, 9000, Ghent, Belgium; National Agricultural research Organization, Plot 11-13 Lugard Avenue, Entebbe- Uganda

**Keywords:** DNA barcodes, Morphology, Morphometrics, Phylogeny, Taxonomy

## Abstract

The new nematode species *Hoplolaimus tuberosus* n. sp., isolated from potato rhizosphere in Budwale sub-county, Mbale district, Eastern Uganda, is characterized based on light and scanning electron microscopy alongside four molecular markers. Females of *H. tuberosus* n. sp. are moderately large (1.2–1.6 mm) and exhibit distinctive morphological features, including an offset lip region with 4–5 lip annuli, a basal lip annule divided into 10–12 irregular blocks, a robust stylet (45–50 μm), a variable lateral field, characterized by one incisure (zigzag longitudinal line formed by anastomoses) anteriorly and posteriorly, and 2–3 irregular, incomplete striae at mid-body, a secretory-excretory pore positioned anterior to the hemizonid, 6 gland nuclei, and a hemispherical to bluntly rounded tail with 8–10 annuli. Males are slightly smaller at 1.0–1.3 mm, have a basal lip annule divided into 2–4 blocks and relatively long spicules (46–58 μm). Phylogenetic analyses of COI mtDNA, ITS-rRNA, 18S-rRNA and D2D3 of 28S-rRNA demonstrated a close relation of the new species with morphologically similar species (*Hoplolaimus columbus*, *Hoplolaimus indicus, Hoplolaimus seinhorsti*, *Hoplolaimus dubius* and *Hoplolaimus pararobustus)* yet *H. tuberosus* n. sp. had in all analyses a distinct phylogenetic position. The population density of 50–75 *H. tuberosus* n. sp. per 100 ml of soil, combined with the polyphagous nature of related *Hoplolaimus* species, suggests that this new species could pose a significant pest threat to potato crops, warranting further pathogenicity studies.

Hoplolaimid species, referred to as lance nematodes belong to the subfamily Hoplolaiminae Filipjev, 1934. They have tulip-shaped stylet knobs, and a large body length (0.9 – 2 mm) (Lewis et al., 1976). Thirty seven (37) species within this genus have been described to date (Handoo and Golden, 1992; Tiwari et al., 2001; Ali et al., 2009; Nguyen et al., 2015; Ma et al., 2019; Ghaderi et al., 2020) of which *H. columbus, H. galeatus*, *H. magnistylus*, and *H. pararobustus* are of economic importance (Fassuliotis, 1974; Nyczepir and Lewis, 1979; Robbins et al., 1987; Robbins et al., 1989; Henn and Dunn, 1989; Fortuner, 1991; Noe, 1993; Koenning et al., 2004; Bae et al., 2008). They feed both endo and ecto-parasitically on plant roots causing damage to various crops including bananas, wheat, chrysanthemums, cotton, pine, corn, alfalfa, beans, peas, cabbage, sweet potatoes, oak, peanuts, sycamore, apple, clover, lawn grasses, okra, maize, rice, sugarcane, sorghum, forage legumes, cowpeas, pigeon peas, rice, tomatoes, palm trees, and ornamental plants (Sher, 1963; Bridge, 1973; Robbins et al., 1987, 1989; Henn and Dunn, 1989; Koenning et al., 2004; Ahmadi Mansourabad, et al., 2016; Holguin et al., 2015; Niere and Karuri, 2018; Mitiku, 2018; Olajide et al., 2023; Bert et al., 2024). There have been reports of lance nematodes parasitizing potatoes (Lima et al., 2018), but no studies have been conducted to confirm their pathogenicity on the crop. Their feeding habits destroy the root system, causing necrosis and reduced growth, and they open wounds through which microorganisms can enter, causing root rot (Khan and Quintanilla, 2003).

Species of this genus have been reported in the USA (Sher 1963; Fassuliotis 1974; Martin et al., 1994; Gazaway and McLean, 2003), Canada, South America, Central America and India (Fortuner, 1991), Iran (Ahmadi Mansourabad et al., 2016), and Africa (Schuurmans and Teunissen, 1938; Marais et al., 2020; Olajide et al., 2023) are among others.

Seven species (*H. pararobustus*, *H. galeatus, H. aegypti, H. seinhorsti, H. columbus H. indicus*, and *H. clarissimus*) have been reported in Africa. *H. pararobustus* is reported in the Democratic Republic of Congo, Angola, Burkina Faso, Cameroon, Dominica, Egypt, Gambia, Guinea, Ivory Coast, Kenya, Madagascar, Malawi, Mozambique, Niger, Nigeria, South Africa, Sri Lanka, Tanzania, Togo, Zimbabwe and Uganda (Schuurmans and Teunissen, 1938; Coyne et al., 2018; Fortuner, 2020; Marais et al., 2020; Olajide et al., 2023). In Uganda, it primarily infests bananas (*Musa* sp.) but also attacks cassava, yam, coffee, and maize (Coyne et al., 2018). *H. galeatus, H. aegypti*, and *H. columbus* are reported in Egypt (Bridge and Starr, 2007; Ibrahim, 1994; Ibrahim et al., 2000). *H. seinhorsti* has been recorded in Nigeria (Bridge, 1973; Bridge and Starr, 2007). Additionally, *H. clarissimus* is found in Egypt (Ibrahim et al., 1994; 2000), while *H. indicus* has been noted in Ghana (Osei et al., 2012).

Species identification of this genus largely has relied on morphological and morphometric characters, such as the number of esophageal gland nuclei, the number of lines in the lateral field, stylet length and body length, the number of labial annuli, the position of scutella along the body, and the hemizonid position with respect to the secretory-excretory (SE) pore (Handoo and Golden, 1992; Bae et al., 2008). However, relying solely on morphology and morphometry for species characterization can be challenging and sometimes impractical, as these methods often struggle to distinguish closely related species with overlapping key characteristics. (Hyman, 1990; Van den Berg et al., 2014). Thus, the use of both morphology and molecular identification methods is indispensable in nematode species identification. Currently, only eleven *Hoplolaimus* species are linked to molecular data, either 18S rRNA, D2-D3 expansion segment of 28S rRNA, ITS rRNA and/or COI mtDNA sequences (Bea et al., 2008; Ma et al., 2011; Ma et al., 2019; Shokoohi et al., 2022; Jumaah et al., 2024).

In the current paper, we describe a new species as *Hoplolaimus tuberosus* n. sp. recovered from a potato field in Eastern Uganda. The dense population of this nematode in the investigated soil samples indicated its potential importance as a potato pest. Nematode characterization was carried out based on morphological information obtained from light microscopy (LM) and scanning electron microscopy (SEM) studies. Illustrations, morphometrics, and phylogenetic analyses of ITS, 18S, and 28S of rDNA and COI of mt DNA are provided.

## Materials and Methods

### Sample collection, soil analyses, nematode extraction and morphological characterization

A survey was conducted to determine the occurrence and distribution of plant-parasitic nematodes on potatoes in Uganda in key potato-growing agroecological zones. Soil and root samples were collected, and nematodes were extracted using the modified Baermann technique (Whitehead and Hemming, 1965). Morphological and morphometric assessment comprised of mounting and examining individual nematodes on temporary slides and then retrieving them for DNA extraction to correlate morphology with molecular data. Morphological vouchers linked with molecular sequences are deposited in BOLD (https://v4.boldsystems.org/index.php/MAS_Management_DataConsole?codes=RQHPL). Other specimens were heat-killed and fixed using a 4% formaldehyde solution and then gradually processed to anhydrous glycerin as per the method described by De Grisse (1969), but the step of nematodes staying overnight in ethanol was skipped. Micrographs of both temporary and permanently fixed specimens were captured, and all measurements were taken from permanently fixed specimens using an Olympus BX50 DIC microscope (Olympus Optical) equipped with an HD Ultra camera. Drawings were created using an Olympus BX51 DIC microscope (Olympus Optical) equipped with a drawing tube. For SEM analysis, 300 mL of freshly prepared fixative solution (4% paraformaldehyde and 1% glutaraldehyde in 0.1 M phosphate buffer, pH 7.3) was transferred into a glass staining block containing nematodes. The block was microwaved for 4 seconds, and the fixed specimens were stored at 4°C for three days. The specimens underwent eight 20-minute dehydration cycles in graded ethanol solutions (30%, 50%, 65%, 75%, 85%, 90%, 95%, and 98%), followed by three 10-minute washes in 100% ethanol. Subsequently, the nematodes were critical point-dried using liquid CO_2_, mounted on stubs with carbon tabs, coated with a 10 nm gold layer (120 seconds at 15 mA), and imaged with a JSM-840 electron microscope at 12 kV. (Singh et al., 2018). The soil texture and organic carbon content was analyzed of the type locality. The organic carbon was determined according to the Walkley and Black method (Walkley and Black, 1934) through oxidation with 1N K2Cr7O7 (7%) in the presence of concentrated H2SO4 (96%), and back-titration using ferrous sulphate using ferroin as indicator. Soil texture was analyzed using the pipette method as outlined by Van Reeuwijk (1993) following removal of organic matter using H_2_O_2_ and carbonates using NaAc.

### Molecular characterization

Nematode DNA was extracted from nematodes recovered from temporarily mounted specimens. The nematodes were washed thrice with distilled water and cut into three pieces using a sterile needle. These pieces were then placed in a PCR tube containing 20μl of worm lysis buffer (50 mM KCl, 10 mM Tris pH 8.3, 2.5 mM MgCl2, 0.45% NP-40 (Tergitol Sigma), 0.45% Tween-20), followed by freezing at −20°C for 10 minutes. After freezing, 1 μl of proteinase K (1.2 mg/ml) was added, and the mixture was incubated at 65°C for 1 hour and then at 95°C for 10 minutes, followed by centrifugation of the lysate at 15,000 g for 1 minute. The DNA samples were stored at −20°C until required for PCR analysis. DNA amplification was conducted using a Bio-Rad T100™ thermocycler with a master mix containing 10μl dH2O, 12.5 μl DreamTaq (Thermo Fisher Scientific), 0.5 μl of each forward and reverse primer (10 μl, and 2 μl of extracted DNA was used. Primers for D2–D3 of 28S amplification were forward primer D2A (5′-ACA AGT ACC GTG AGG GAA AGT TG-3′) and the reverse primer D3B (5′-TCC TCG GAA GGA ACC AGC TAC TA-3′) (Nunn, 1992). PCR conditions were pre-denaturation at 94°C for 4 min followed by 40 cycles of 94°C for 1 min, 52°C for 1 min, and 72°C for 1 min and finished with one cycle at 72°C for 10 min. For the internal transcribed spacer (ITS) region, TW81 (5′-GTTTCCGTAGGTGAACCTGC-3′) and AB28 (5′-ATTGCTTAAGTTCAGCGGGT-3′) primers were used (Joyce et al., 1994). The PCR conditions were pre-denaturation at 94°C for 4 min followed by 40 cycles of 94°C for 1 min, 57°C for 1 min, 72°C for 1 min and finished with one cycle at 72°C for 10 min. For COI, the primers used were JB3: 5′-TTT TTT GGG CAT CCT GAG GTT TAT-3′ and JB4.5: 5′-TAA AGA AAG AAC ATA ATG AAA ATG-3′ (Bowles et al., 1992) and the PCR conditions were 1 min at 94°C, 1min at 45°C, and 1 min at 72°C for 38 cycles. The primers SSU18A: 5′-AAAGATTAAGCCATGCATG-3′ and SSU26R: 5′-CATTCTTGGCAAATGCTTTCG-3′ (Mayer et al., 2007) were used to amplify the 18S gene following thermal profiles described in Singh et al. (2018). PCR products were purified enzymatically, as detailed by Singh et al. (2020), and sent for sequencing in both forward and reverse directions to Macrogen (https://dna.macrogen.com). Contigs from the sequences were assembled using Geneious Prime software, version 2024.0.7 and deposited in GenBank.

### Phylogenetic analysis

The acquired sequences were analyzed along with sequence data sets of other *Hoplolaimus* species available in GenBank. Multiple sequence alignments were conducted using MUSCLE with default settings, followed by manual trimming of the poorly aligned ends. Phylogenetic analysis using Bayesian inference (BI) was performed with MrBayes 3.2.6 (Huelsenbeck et al., 2001) under the GTR + I + G nucleotide substitution model. The analysis was executed for 1 × 10^6^ generations across four separate runs, with a burn-in of 20% and a subsampling frequency of 200 generations (Huelsenbeck et al., 2001). The resulting phylogenetic trees were visualized using Tree View, including the posterior probability (PP) values for the relevant clades. All phylogenetic programs were performed within Geneious Prime, version 2024.0.7.

## Results

### Survey information

Soil samples collected from two distinct potato fields; BUD1 (1°03′48.636″N, 34°15′19.404″E) and BUD5 (1°03′36.5394″N, 34°14′55.0674″E) in Budwale Sub-county, Mbale District, Eastern Uganda, revealed the presence of several nematode species, including an undescribed species belonging to the genus *Hoplolaimus* Daday, 1905. The BUD5 soil sample (the type locality) was characterized by a fine-textured clay soil with high clay content, low sand content, and moderate silt content (72% clay; 24% silt; 8% sand) and an organic content of 2.6%. Up to 50 – 75 specimens per 100 ml of soil were retrieved including juveniles, males, and females. The two fields were previously utilized for potato and bean cultivation in the two preceding growing seasons. This region is characterized by a humid subtropical climate, 15°C and 28°C temperature ranges, and a bi-modal precipitation pattern, with an average annual rainfall of 1500 mm (Jiang et al., 2014).

### Systematics

**Figure 1:**
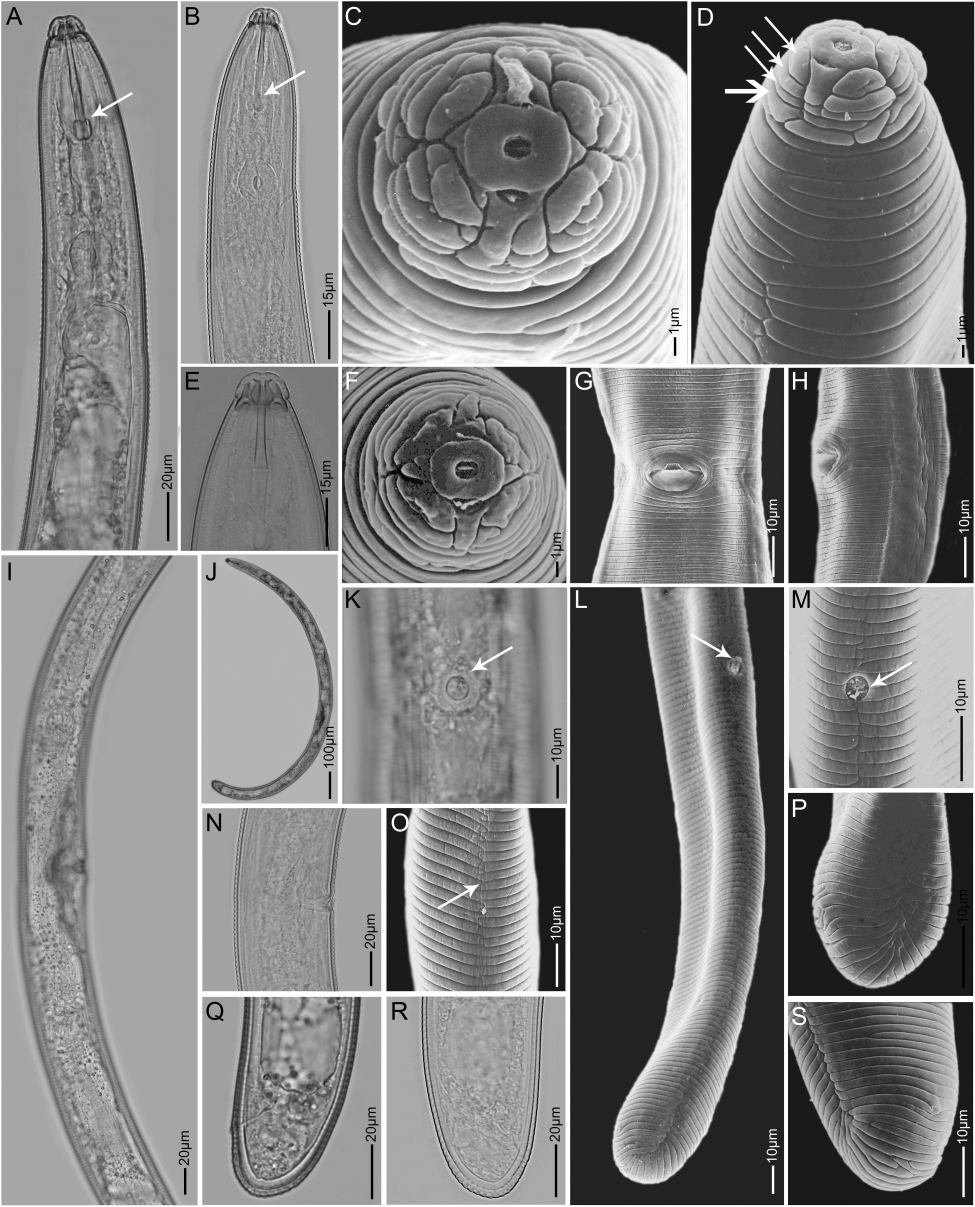
Light- and scanning electron microscopy images of females of *Hoplolaimus tuberosus* n. sp. A, B, E: Anterior body in lateral view showing the position of the SE pore opening and the hemizonid, stylet and stylet knobs, and median bulb, (arrows indicate tulip-shaped stylet knobs); C, D, F: Head region showing lip annuli, oral disc and longitudinal striations on the basal lip annule, (arrows indicate the four lip annuli, horizontal arrow the basal lip annule); G, H, I, N: Vulva region in ventral and lateral views and the out stretched anterior and posterior gonads; J: Body habitus; K, L, M: Scutella (appointed with arrow), lateral view; O: Mid body lateral field, arrow showing zigzag longitudinal line formed by anastomoses; P, S: Lateral field in the posterior region; Q, R: Tail region showing anal opening and tail annuli.

**Figure 2:**
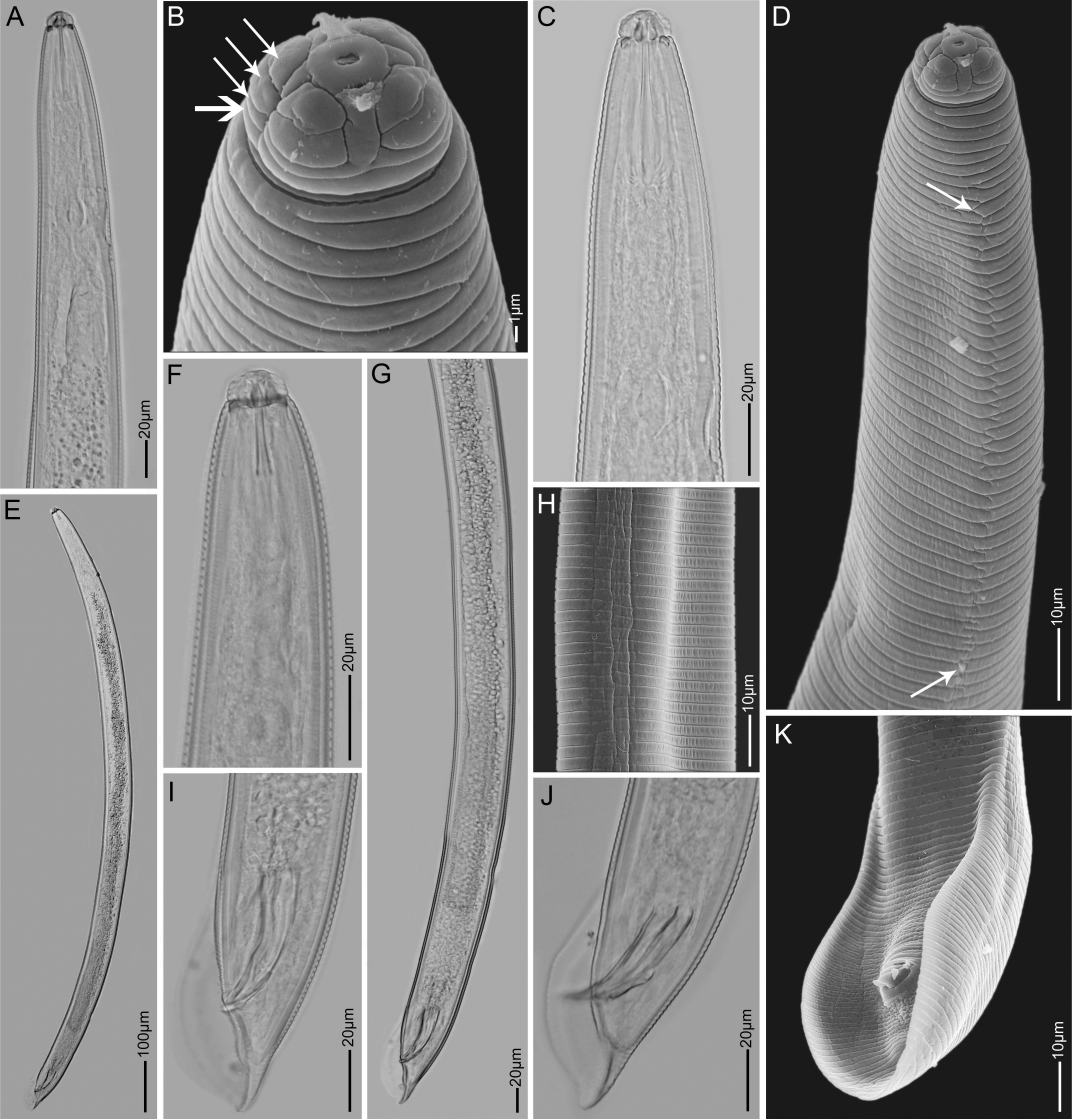
Light- and scanning electron microscopy images of *Hoplolaimus tuberous* n. sp. males. A, B, C, D and F: Anterior body in lateral view showing head region, lip annuli (arrows; horizontal arrow indicates the basal lip annule), stylet, medium bulb and the lateral field (arrow indicate zigzag longitudinal line formed by anastomoses); E: body habitus; H: Mid body lateral field; G, I, J, K: Lateral and ventral view of the tail region showing spicules and bursa.

**Figure 3:**
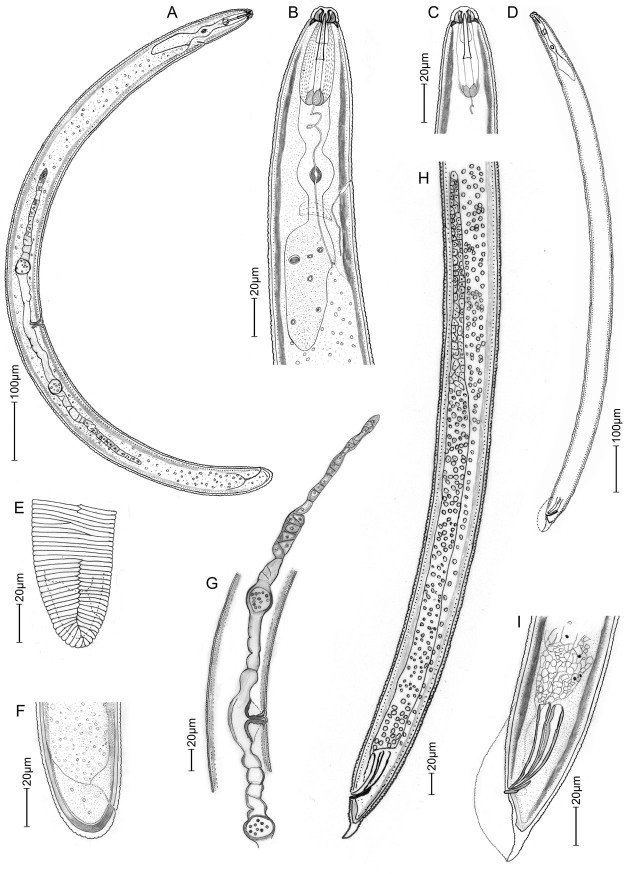
Illustrations of *Hoplolaimus tuberosus* n. sp.: A: Female whole body; B: Female anterior region; C: Male anterior region; D: Male whole body; E, F: Female tail region; G: Female reproductive system; H: Male reproductive system; I: Male spicule, tail, and bursa.

*Hoplolaimus tuberosus* n. sp. (Figs. 1–3; Table 1).

**Table 1:** Morphometrics of *Hoplolaimus tuberosus* n. sp. specimens from Budwale subcounty Eastern Uganda (GPS location: 1° 3′ 36.5394″N; 34° 14′ 55.0674″E).

**Character**	**Holotype**	**Female paratypes**	**Male paratypes**
n	1	18	11
L	1273	1439 ± 146 (1211–1629)	1216 ± 92 (1048–1343)
a	22	22 ± 1.2 (20.7–23.8)	23.6 ± 1.5 (22–27.3)
b	10	10.2 ± 1.1 (8.6–12.3)	9 ± 0.8 (8.2–8.4)
b′	7	8 ± 0.61 (6.2–9)	7 ± 0.6 (6.1–8)
c	93.5	99.8 ± 10 (85–122)	30 ± 2.3 (25–33)
c′	0.42	0.44 ± 0.04 (0.36–0.6)	1.6 ± 0.1 (1.4–1.8)
V	724	823 ± 82 (697–1013)	
V(%)	57	57 ± 1.06 (55–59.5)	
Max. body diam	58	64 ± 4 (58–70)	51.5 ± 4 (45–60)
Diameter at mid body	56	60 ± 5 (50–66)	50 ± 4 (43.5–58)
Lip annuli	4–5	4–5	4
Lip height	8.3	7.5 ± 0.7 (6.7–9)	8 ± 0.5 (7–8.3)
Lip diam.	15.7	16.5 ± 1.1 (15–18)	15 ± 0.6 (14.2–16.4)
Conus	25.5	25 ± 1 (24–26)	23.6 ± 0.6 (22.3–24.2)
Stylet length	49	48 ± 1.6 (45.4–51)	45.6 ± 1.3 (43–47.3)
Knob diam.	7.7	8.6 ± 0.86 (6.8–10)	7.5 ± 1 (6.7–9)
Knob height	7	6.6 ± 0.8 (5–7.5)	6 ± 0.2 (5.6–6.2)
Stylet knobs to end of pharyngeal gland	131	147 ± 10 (130–163)	137 ± 10 (126–154)
Pharynx & glands (anterior to pharyngeal gland tip)	184	184 ± 17 (153–221)	178 ± 17.7 (142–203)
Tail length	13.6	14 ± 1.07 (11.3–16)	41 ± 3.5 (45.6–46)
Anal body diam.	32	32 ± 1.3 (30–36)	26 ± 2 (22.5–29)
Excretory pore (from anterior end)	93	113 ± 10 (93–125)	104 ± 12 (94–121)
DGO	6.3	7 ± 0.5 (5.9–8)	6 ± 0.4 (5.6–6.6)
Spicule length			52 ± 3.6 (46–58)
Gubernaculum			22.6 ± 2 (21.5–27)
Annulus diam.	2.5	2.7 ± 0.3 (2.1–3)	2.5 ± 0.2 (2.2–3)

### Description

#### Females (n = 18)

Body large (1.2–1.6 mm), C-shaped when relaxed, vermiform and tapering slightly at ends. Lip region offset; 7–9 μm high and 15–18 μm wide, with 4–5 clearly marked lip annuli. Basal lip annule divided into 10–12 irregular/unequal blocks (Fig. 1D). Stylet massive, long and robust (45–51 μm) (Table 1), with tulip-shaped basal knobs. Dorsal pharyngeal gland opening near stylet base (14.5% of spear length). Pharyngeal glands overlap intestine dorsally and laterally, gland nuclei duplicated to a total of six nuclei. Pharyngeal-intestinal junction at 9–10% from anterior end. Excretory pore prominent, 93–124 μm from anterior end and located opposite the median bulb, at about 8% of body length. Cuticle annulated, mid-body annuli measuring about 2.5–3 μm. Lateral field variable represented by 1 incisure (longitudinal zigzag line) resulting from characteristic anastomoses at anterior and posterior ends, and 2–3 incomplete incisures, irregularly scattered at mid body (Figs. 1D, 1S, 3E). Hemizonid large, about 12 annuli posterior to the excretory pore extending over 2 annuli. Reproductive system didelphic-amphidelphic with both genital branches outstretched and sometimes masked by intestinal globules. Vulva at 57% from anterior end, with distinct epiptygma. Spermatheca present with sperms. Phasmids enlarged to oval shaped scutella, one anterior and one posterior of the vulva. Right phasmid located anterior to the vulva at 30–40% and the left posterior at 60–85% from anterior end. Tail 14–21 μm long with 8–10 annuli. Terminus hemispherical to bluntly rounded.

#### Males (n = 11)

Body J-shaped when relaxed, slightly slender and shorter than females. Lip region set off, the labial region with four annuli. Basal lip annule divided into 2–4 blocks (Fig. 2B). One testis outstretched anteriorly. Spicules long and well developed, measuring 46–58 μm, arcuate with distal flanges. Gubernaculum large and protrusible. Bursa extending to tail tip.

### Diagnosis and relationships

*Hoplolaimus tuberosus* n. sp. is characterized by moderately large sized females (1.2–1.6 mm); a robust stylet averaging 48 μm long; an offset head with 4–5 annuli, a tessellated basal lip annule forming 10–12 irregular blocks; hemizonid located twelve annuli posterior to excretory pore; a variable lateral field represented by 1 incisure (longitudinal zigzag line formed by anastomoses) at the anterior and posterior end and 2–3 irregular and incomplete striae at mid-body; a pharyngeal gland with six gland nuclei; and a short tail measuring 11–16 μm with 8–10 annuli. Males are present and slightly smaller than females; their basal lip annule divided into 2–4 longitudinal striae; and long spicules measuring 46–58 μm.

Using the *Hoplolaimus* key and compendium provided by Handoo and Golden (1992) along with recent descriptions, *Hoplolaimus tuberosus* n. sp. is found to be morphologically close to *H. indicus* Sher, 1963, *H. dubius* Chaturvedi and Khera, 1979, *H. aegypti* Shafiee and Koura, 1969, *H. seinhorsti* Luc, 1958, *H. columbus* Sher, 1963 and *H. pararobustus* Schuurmans Stekhoven and Teunissen, 1938, by having excretory pore anterior to hemizonid, lateral field formed by one incisure, one scutellum anterior and one posterior to the vulva and six gland nuclei (except *H. pararobustus*) (Table 2). It differs from all by having fewer tail annuli; 8–10 vs 17–27 for *H. aegypti,* 10–15 for *H. seinhorsti,* 8–22 for *H. indicus,* 16–22 for *H. columbus,* 7–15 for *H. pararobustus* and 10–15 for *H. dubius*. In addition, *Hoplolaimus tuberosus* n. sp. differs from *H. indicus* by the relatively longer stylet (45–51 μm vs 33–47 μm), different number of longitudinal striae on the basal lip annule (10–12 vs 6–20) and the longer spicules (46–58 μm vs 34–42 μm); from *H. seinhorsti* by the relatively longer stylet (45–51 μm vs 40–49 μm), and males (present vs unknown); from *H. columbus* by the relatively longer stylet (45–51 μm vs 40–48 μm) and males (present vs. rarely present); from *H. aegypti* by having shorter spicules (46–58 μm vs 54–65 μm); from *H. dubius* by the relatively longer body length (1.2–1.6 mm vs 1.05–1.27 mm in females), longer stylet (45–51 μm vs 31–42μm), and longer spicules (46–58 μm vs 37–44 um); and from *H. pararobustus* by more esophageal gland nuclei (6 vs 3), less longitudinal striae on the basal lip annule (10–12 vs 18–25), and a relatively longer stylet (45–51 μm vs 38–49 μm). It also clearly differs molecularly from all these species based on all the genes analyzed.

**Table 2:** Comparison of *Hoplolaimus tuberosus* n. sp. with seven *Hoplolaimus* species reported from Africa, focusing on some key female characteristics and the presence of males.

**Species**	**Body length (mm)**	**Longitudinal striae on basal lip annuli**	**Lip Annuli Number**	**Lateral incisures**	**Stylet length**	**Oesophageal gland nuclei**	**Excretory pore in relation to hemizonid**	**Phasmid in relation to Vulva**	**Tail annuli**	**Males**	**Spicule length**
*Hoplolaimus tuberos* n. sp.	1.2–1.8	10–12	4 (rarely 3or 5)	1 (2–3 incomplete)	45–51	6	Anterior	One anterior and one posterior	8–10	Present	46–58
*H. pararobustus*	0.95 –1.7	18–35	4–5	1 (2–3)	38–49	3	Anterior	One anterior and one posterior	7–15	Present	40–57
*H. galeatus*	1.2–1.9	32–36	5	4	43–52	3	Posterior	One anterior and one posterior	10–16	Present	40–52
*H. seinhorsti*	1–1.6	8–12	4	1	40–49	6	Anterior	One anterior and one posterior	10–15	Unknown	
*H. aegypti*	1.3–1.9	13–22	4	1	45–50	5 (6th obscure)	Anterior	One anterior and one posterior	17–27	Present	54–65
*H. columbus*	1.3–1.8	10–15	3	1	40–48	6	Anterior	One anterior and one posterior	16–22	Rarely present	36.6–52.5
*H. clarissimus*	1.4–1.8	18–31	4 (rarely 3or 5)	4	46–52.5	6	Posterior	One anterior and one posterior	20–26	Present	58.5 (55.5–61.5)
*H. indicus*	1.1–1.6	6–20	3–4	1 or (2–3 incomplete)	33–47	6	Anterior	One anterior and one posterior	14 (8–22)	Present	34–42

### Etymology

*Hoplolaimus tuberosus* n. sp. was isolated from soil collected from the rhizosphere of potatoes (*Solanum tuberosum*) and the species epithet *tuberosus* is derived from the species epithet of the crop's name.

### Type of locality and habitat

Type material was obtained from Budwale subcounty (field BUD5) (GPS location: 1° 3′ 36.5394″N; 34° 14′ 55.0674″E, elevation 1956 m a.s.l.), Mbale district, Eastern Uganda, associated with potatoes. The new species was also found in another field (BUD1), about 2 km away from the type locality, in the same sub-county. The conspecificity of both populations was supported by 99.5–100% identical sequences.

### Type designation and deposition

Female holotype (UGMD_104479), 2 females (UGMD_104480), 1 male and 2 females (UGMD_104481), 1 male (UGMD_104482), and 3 male paratypes (UGMD_104483) are deposited at Ghent University Museum, Zoology Collections, Belgium. Six additional slides containing four female paratypes and 3 male paratypes are deposited at UGent Nematode Collection of Nematology Research Unit of Ghent University, Belgium (UGnem349-354). Two slides, one containing two female paratypes and one with two male paratypes are deposited at Makerere University, Nematology Research Unit, Uganda. The video (virtual microscopy) of the holotype is available on https://nematodes.ugent.be/vce.html. Registered in ZooBank (http://zoobank.org/) with identifier: urn:lsid:zoobank.org:pub:2C7F5543-EDEA-4B6A-944B-7CE02C2B5ACA.

### Molecular characterization and phylogenetic relationships

**Figure 4:**
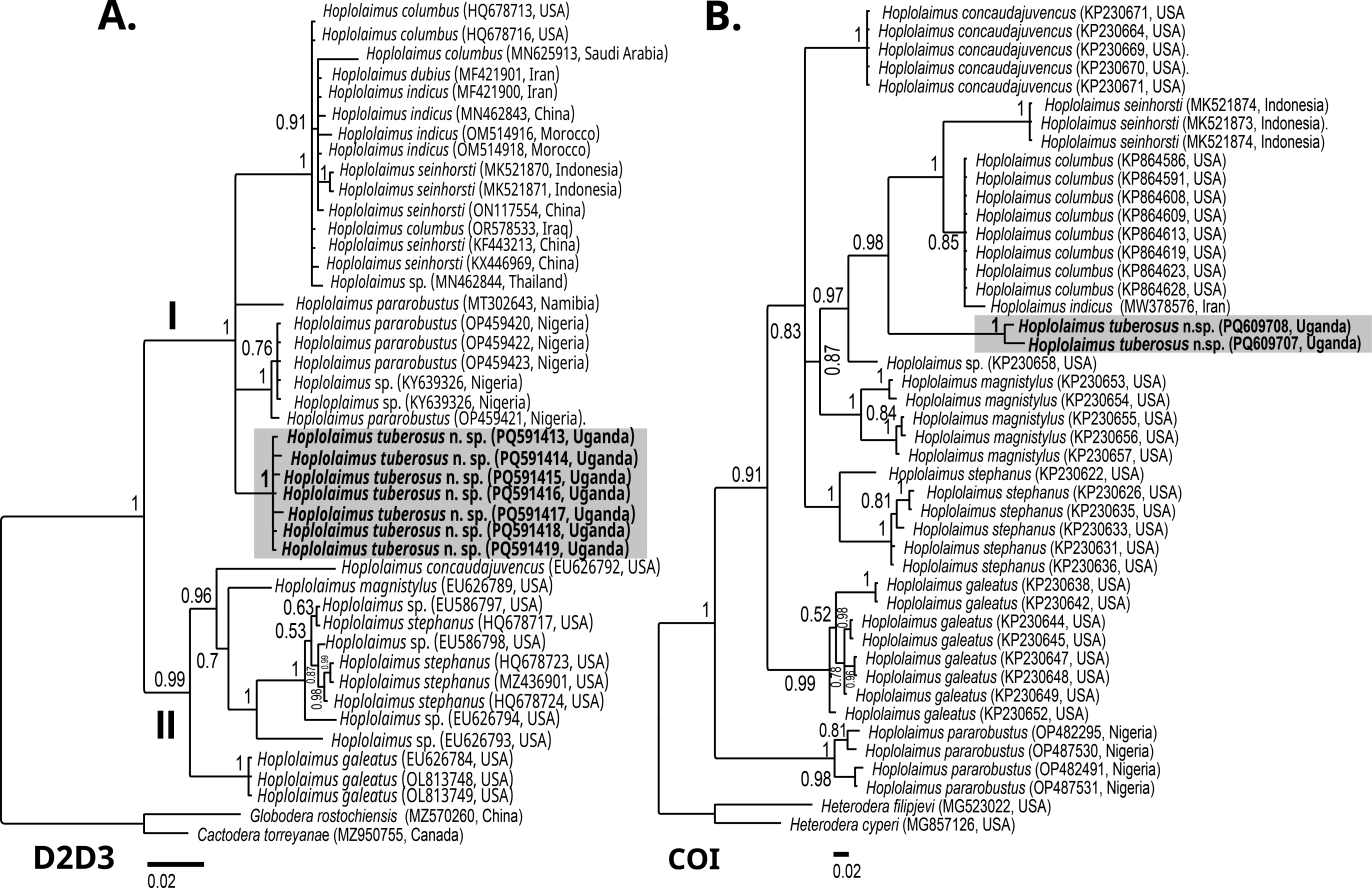
BI phylogenetic tree generated from the analysis of D2–D3 of 28S rRNA (A) and COI of mt DNA (B) sequences using GTR + G + I nucleotide substitution model. Bayesian posterior probabilities are given next to each node and sequences of *Hoplolaimus tuberosus* n. sp. are in bold. Clade numbers are taken from Olajide et al. (2023).

The amplification of the D2–D3 expansion segment of the 28S-rRNA, 18S-rRNA, ITS-rRNA, and COI of mtDNA genes produced fragments approximately 720, 890, 986, and 417 bp in length, respectively. The seven newly obtained sequences of the D2–D3 region from male and female specimens showed intraspecific variations of 0–4 bp, with a similarity of 99.5% to 100%. A phylogenetic tree including eight different species and seven unidentified *Hoplolaimus* sequences (Fig. 4A) revealed a maximally supported clade of *H. tuberosus* n. sp. and sequences of *H. pararobustus*, *H. columbus*, *H. indicus*, *H. seinhorsti*, and *H. dubius* (Clade 1)*. H. tuberosus* n. sp. is in an unresolved position from these species, but the new sequences differ by 22–26 bp (3.6%), 25–35 bp (5%), 33 bp (5.4%), 31–55 bp (6%), and 33–42 bp (5.4–7.8%). Thus, the highest observed similarity was only 96.7%, specifically with *H. pararobustus* sequences (OP459422, OP459421, and OP459423) reported from Nigeria (Olajide et al., 2023).

The obtained COI mtDNA sequences displayed an intraspecific variation of 13 nucleotides (95.7% similarity) and formed a well-supported sister relationship (PP: 0.98) with *H. columbus*, *H. indicus*, and *H. seinhorsti* (Fig. 4B). These sequences differed significantly from the aforementioned species by 56–57 bp (16–18%), 61 bp (18%), and 64–87 bp (21–25%), respectively. The closest match was *H. columbus* (KP864609) with a similarity of only 83.8% (Holguin et al., 2015).

**Figure 5:**
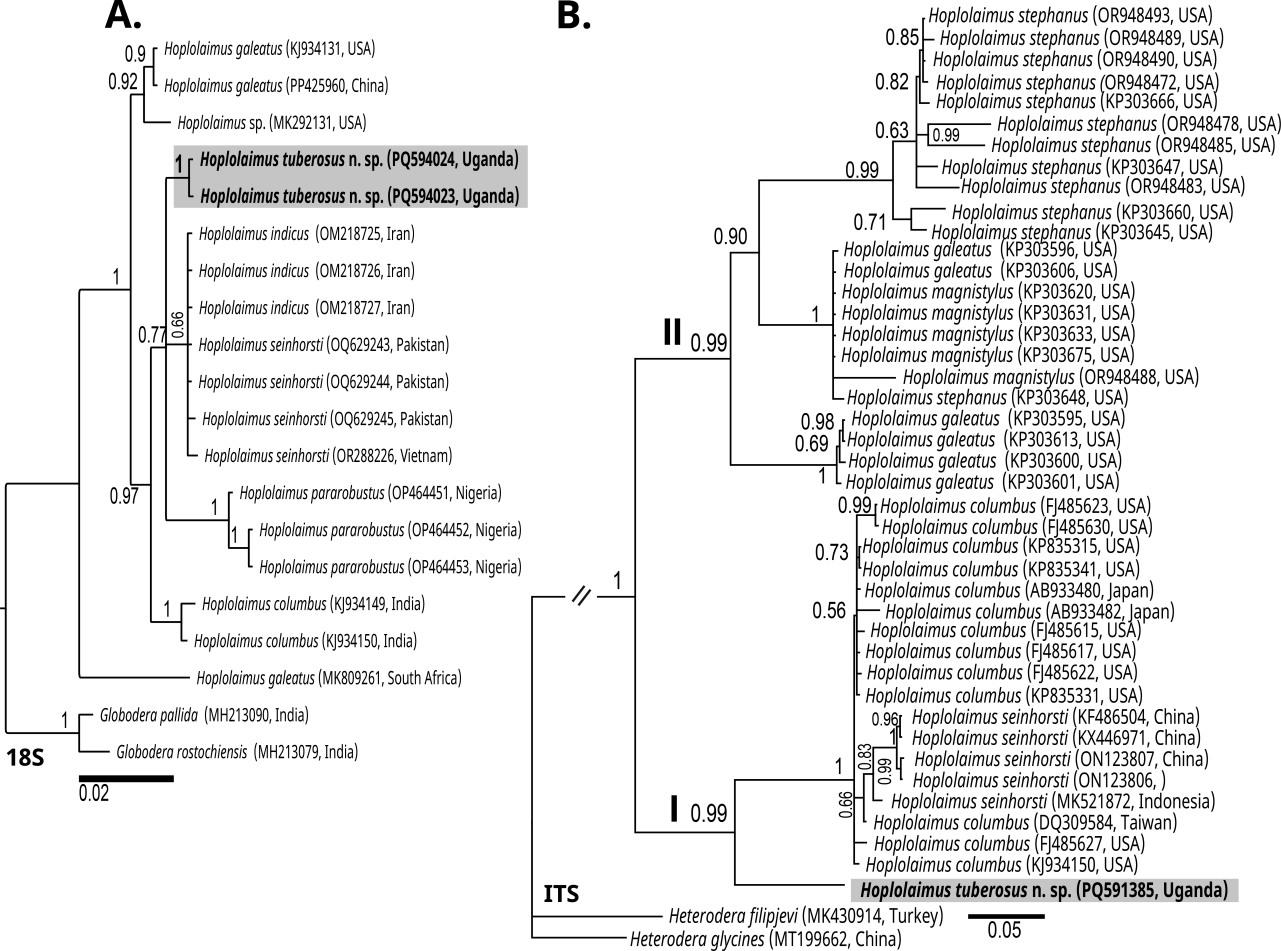
BI phylogenetic tree generated from the analysis of 18S-rRNA (A) and ITS-rRNA (B) sequences using GTR + G + I nucleotide substitution model. Bayesian posterior probabilities are given next to each node and sequences of *Hoplolaimus tuberosus* n. sp. are in bold. Clade numbers are taken from Olajide et al. (2023).

The two obtained 18S rDNA sequences of *H. tuberosus* n. sp. (Fig. 5A) showed 99.9% similarity, differing by only 1 bp. In the resulting phylogeny, the sequences formed a well-supported clade (PP = 0.97) together with *H. indicus*, *H. seinhorsti*, *H. columbus*, and *H. pararobustus*, showing differences of 2–6 bp (0.2–0.7%), 4–16 bp (1.4–5%), 9–19 bp (1–2%), and 17–24 bp (2–2.4%), respectively.

Finally, ITS sequence analysis revealed a maximally supported sister relationship with *H. columbus* and *H. seinhorsti* (Fig. 5B; clade 1), showing differences of 55–182 bp (12.6–19.2%) and 174–185 bp (19%), respectively. The most similar sequence was identified as *H. columbus* (AB933482), with only 87.4% similarity.

## Discussion

This study describes *Hoplolaimus tuberosus* n. sp., a newly identified species of *Hoplolaimus*, distinguished from known species through a combination of molecular and morphological analyses. Analyses of the D2–D3 expansion segments of 28S, ITS, 18S-rRNA, and COI genes revealed significant interspecific sequence differences and a unique phylogenetic position, confirming the distinctiveness of *H. tuberosus* n. sp. Phylogenetic analysis places *H. tuberosus* n. sp. in close relation to morphologically similar species, including *H. columbus*, *H. seinhorsti*, *H. indicus*, and *H. pararobustus*. Morphologically, *H. tuberosus* n. sp. is characterized by fewer tail ventral annulations in females and relatively longer spicules in males compared to these related species. However, most diagnostic features are consistent among these species, reflecting congruence between molecular and morphological similarities. These subtle morphological distinctions underscore the importance of integrating morphological traits with DNA barcoding for accurate nematode identification and characterization (Subbotin, 2015).

While this study has not yet confirmed whether *H. tuberosus* n. sp. specifically parasitizes potatoes, previous reports of *Hoplolaimus* spp. in potato farms (El-Saedy et al., 2018), along with the observed population density of 50–75 *H. tuberosus* n. sp. per 100 ml of soil and the polyphagous nature of related *Hoplolaimus* species, suggest its potential as a potato pest. Moreover, the damage threshold for *H. columbus* is estimated at 50 individuals per 100 ml of soil (Lewis et al., 1993), implying that *H. tuberosus* n. sp. may pose a similar risk based on its population density.

Given the vital role of potatoes as a staple crop for food security and income generation, further research is crucial to evaluate the pathogenicity of this nematode and its potential quantitative and qualitative impacts on potato production. Such studies are necessary to establish damage thresholds, quantify potential yield losses, and develop effective management strategies to mitigate its effects.
